# The Adverse Effects of Air Pollution on the Eye: A Review

**DOI:** 10.3390/ijerph19031186

**Published:** 2022-01-21

**Authors:** Chia-Ching Lin, Chien-Chih Chiu, Po-Yen Lee, Kuo-Jen Chen, Chen-Xi He, Sheng-Kai Hsu, Kai-Chun Cheng

**Affiliations:** 1 Department of Ophthalmology, Kaohsiung Municipal Siaogang Hospital, Kaohsiung 807, Taiwan; somepp322@gmail.com (C.-C.L.); 0870649@kmhk.org.tw (K.-J.C.); 2 Department of Ophthalmology, Kaohsiung Medical University Hospital, Kaohsiung 807, Taiwan; maco69@gmail.com; 3 Department of Biotechnology, Kaohsiung Medical University, Kaohsiung 807, Taiwan; cchiu@kmu.edu.tw (C.-C.C.); u106500025@kmu.edu.tw (C.-X.H.); b043100050@gmail.com (S.-K.H.); 4 Department of Biological Sciences, National Sun Yat-sen University, Kaohsiung 804, Taiwan; 5 Graduate Institute of Medicine, College of Medicine, Kaohsiung Medical University, Kaohsiung 807, Taiwan; 6 Department of Ophthalmology, School of Medicine, College of Medicine, Kaohsiung Medical University, Kaohsiung 812, Taiwan

**Keywords:** air pollution, eye diseases, ocular surface diseases, glaucoma, cataract, retinal diseases

## Abstract

Air pollution is inevitably the result of human civilization, industrialization, and globalization. It is composed of a mixture of gases and particles at harmful levels. Particulate matter (PM), nitrogen oxides (NOx), and carbon dioxides (CO_2_) are mainly generated from vehicle emissions and fuel consumption and are the main materials causing outdoor air pollution. Exposure to polluted outdoor air has been proven to be harmful to human eyes. On the other hand, indoor air pollution from environmental tobacco smoking, heating, cooking, or poor indoor ventilation is also related to several eye diseases, including conjunctivitis, glaucoma, cataracts, and age-related macular degeneration (AMD). In the past 30 years, no updated review has provided an overview of the impact of air pollution on the eye. We reviewed reports on air pollution and eye diseases in the last three decades in the PubMed database, Medline databases, and Google Scholar and discussed the effect of various outdoor and indoor pollutants on human eyes.

## 1. Introduction

Air is essential for the survival and development of all lives on Earth. Its quality directly influences human health and is closely affected by the extent of civilization. Air pollution is a major contributor to the global burden of disease. There was increased mortality related to air pollution in both developing countries and developed countries such as the United States, despite more rigorous air quality standards [1,2,3]. Although there are many natural sources of air pollution, for example, wildfires and volcanoes, since the Industrial Revolution, human technology has distributed the majority of substantial air pollution. The evolution of human civilization has been accompanied by industrialization and global transportation. Due to the development of industrialization, increasing numbers of fuel-burning motorized vehicles and factories have resulted in high levels of air pollution and poor air quality. For example, according to the Central Weather Bureau of Taiwan, the air quality index (AQI) in south Taiwan, where the main power plants of this island were located, was between 130~160 in January 2021, which is considered harmful to the normal population, and outdoor activities were not suggested.

### 1.1. The Composition of Air Pollution

Air pollution comprises a complex mixture of gas-phase pollutants and particles at harmful levels that are disbursed into the atmosphere due to either natural or human activities [4]. There are many pollutants in the atmosphere, such as sulfur dioxide (SO_2_), nitrogen dioxide (NO_2_), carbon dioxide (CO_2_), nitrogen monoxide (NO), carbon monoxide (CO), nitrogen oxides (NOx), particulate matter 2.5 (PM_2.5_), and particulate matter 10 (PM_10_), mostly generated from burning fuels or industrial production. In addition to traffic and industry activities, daily life activities, including tobacco smoking, household decorating and cooking, also produce COx, NOx, and volatile organic compounds (VOCs). For example, formaldehyde can cause DNA damage in animal cells, and its carcinogenicity has been assessed by many studies in the past three decades [5].

Indoor activities with poor ventilation of buildings in modern life are another cause of health problems. There is an increasing prevalence of asthma, autism, and childhood cancer with everyday exposure to these indoor chemical pollutants [6].

### 1.2. The Influences of Air Pollution to Human Eyes

Air pollution influences daily living in societies and even jeopardizes the survival of humans. It is widely known that outdoor air pollution influences health. Air pollution induces many health problems and diseases, such as cardiovascular disorders, respiratory tract problems, ocular disease, neurologic disease, cancer, and death [7,8,9,10].

The cornea is the most sensitive structure in the human body due to numerous innervations in the ocular surface and thus is extremely sensitive to environmental agents [11,12]. The eyes defend against potentially harmful external material with only a thin layer of precorneal tear film; as a result, human eyes are susceptible to the adverse effects of air pollution [13].

The adverse effects of air pollutants such as CO, NOx, PM, and O_3_ on human eyes consist of mostly irritation and inflammation, with conjunctivitis being a frequent problem [14]. Numerous studies have tried to determine the impact of environmental toxins on the ocular surface. Saxena and colleagues found that persons who traveled to highly polluted areas where the PM level was five times higher than the WHO annual average limit of 60 µg/m^3^ suffered from extensive subclinical ocular surface changes [10]. Versura and associates reported that the mixture of air pollutants led to cytological changes and inflammation in the ocular surface, contributing to eye discomfort [15]. An increasing number of studies have indicated that air pollutants such as PM_2.5_ are associated with allergic conjunctivitis [16,17] and glaucoma [18,19], and exposure to tobacco smoke can cause cataracts [20]. Moreover, age-related macular degeneration (AMD) is related to exposure to traffic-related air pollutants [21].

Many research investigations have surveyed the association between air quality and outpatient or emergency room visits with respect to respiratory or cardiovascular symptoms. However, only a limited number of studies have investigated the relationship between air pollution and ocular diseases. In the past three decades, no updated review has provided an overview of the impact of air pollution on the eye. This article aimed to determine the significance of the influence of air pollution on the eye and will discuss the effects of various outdoor and indoor pollutants on human eyes by reviewing papers in the last three decades.

## 2. Material and Methods

The authors performed a review of the literature using PubMed databases, Medline databases and Google Scholar from 1 January 1990, to 31 July 2021. The main inclusion criterion for this review was data on ocular diseases associated with air pollution. The following keywords were used to search the databases: environmental pollution, air pollution, eye disease, ocular disease, acute effect, chronic or long-term effect, ocular surface, inflammation, conjunctivitis, cornea, dry eye, cataract, glaucoma, retinal disease, and retinal vascular disease. Health issues about moisture damage and microbiology-related topics were excluded from this overview. To provide the most up-to-date evidence, we preferred to choose more recent articles that were published in the last five years. The results were restricted to publications in English. Totally, 28 review papers and 83 research papers were cited in the review paper.

## 3. Results and Discussion

There are multitudes of studies elucidating that environmental pollution is harmful to the health of human organ systems, including the skin, oropharyngeal, and respiratory systems [1,2,7,8,9,10]. Eyes, as the most important sensory organs, are inevitably the first to be affected by air pollution. Therefore, in the past three decades, the number of studies evaluating the harmful effects of air pollution on the eye has increased.

**Figure 1:**
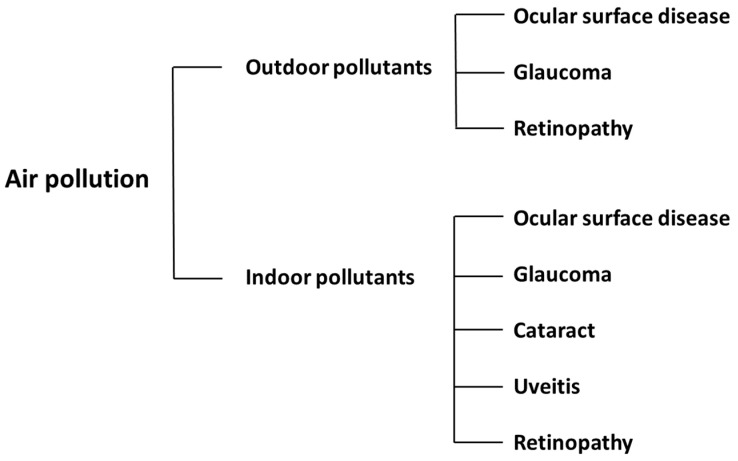
The impact of air pollutants on eye diseases from outdoor and indoor sources.

Our analysis of the relevant studies included in this narrative review (see Table 1) demonstrated that the most common ophthalmologic disorder related to air pollution was inflammation of the conjunctiva (conjunctivitis). Figure 1 illustrates the outline of this article and the impact of air pollution on the eye. Here, we discuss each adverse effect of air pollution according to the origin of the compound.

### 3.1. Outdoor Air Pollution

Outdoor air pollution is a significant public health problem in population centers worldwide. For decades, studies have demonstrated a strong association between air pollution and a spectrum of ill health effects. Outdoor air pollution is a major environmental health hazard that was associated with 3.7 million deaths worldwide in 2012 [46] and 4.2 million deaths in 2016 [47]. The increasing trend in attributable deaths from 1990 to 2015 was partially due to the increase in outdoor air pollution levels in low- and middle-income countries [1]. In 2016, the International Agency for Research on Cancer (IARC) classified outdoor air pollution as a human carcinogen, according to adequate evidence, especially for lung cancer [48]. Automobile traffic is the predominant source of outdoor air pollution in developed urban areas. The components of outdoor air pollution are complicated and dynamic and include ozone (O_3_), nitrogen dioxide (NO_2_), sulfur dioxide (SO_2_), lead (Pb), carbon monoxide (CO), and particulate matter (PM). The components vary seasonally and are affected by human activity and climatic events [49]. PM is further classified into coarse PM (PM_10_, PM with an aerodynamic diameter ≤ 10 μm), fine PM (PM_2.5_, PM with an aerodynamic diameter ≤ 2.5 μm), and ultrafine PM (PM_1.0_, PM with an aerodynamic diameter ≤ 1.0 μm) [50].

Pollutants originate from different sources. For example, NO_2_ and ground-level O_3_ (which derives from the effect of ultraviolet light on nitrogen dioxide) are primarily emitted from vehicle exhaust systems, while SO_2_ originates from the burning of sulfur-containing fuels (e.g., coal-burning plants). Coarse PM primarily results from scattered ground or airborne dust; fine and ultrafine PM derive primarily from vehicular exhaust [51]. Coarse and fine particulates differ in not only their physical properties and size but also their chemical components. PM_10_ primarily consists of geological materials, in comparison to PM_2.5_ and PM_1.0_, which have larger fragments of elemental and organic carbon [52]. These variations in PM chemical composition are related to different toxicity profiles and can be used as tracers of vehicular emanations. For example, elemental carbon can be used to track traffic-related emissions [53]. Outdoor air pollution consists of both primary pollutants released directly into the atmosphere and secondary pollutants formed in the air due to chemical transformation of primary pollutants. These chemical reactions are affected by temperature and thus can be affected by global climate warming. In addition, accumulated evidence has implied that air pollution can not only exacerbate ocular symptoms but also cause new-onset ocular disease. Table 2 demonstrates the 2021 World Health Organization (WHO) air quality guidelines, which were tightened following recommendations in 2005. According to the UN organization, the new recommendations reflect the recent evidence of the significantly higher-than-thought impact of even lower concentrations of air pollution on human health and wellbeing. A recent study estimated the death toll of air pollution at 8.7 million per year [54].

#### 3.1.1. Ocular Surface Diseases (OSDs)

Traffic- and industry-related airborne byproducts account for most outdoor air pollution. For example, in New Delhi, India, the transportation component of air pollution was 72%, and the industry component was 20% in 2003. The level of suspended particulate matter (SPM) in New Delhi was five times higher than the annual average control limit of 60 mg/m^3^ set by the World Health Organization (WHO). One study showed that people in New Delhi who commuted daily to the workplace using open vehicles (e.g., scooters, motorcycles, or bicycles) for more than 10 years had more ocular surface symptoms, such as redness, irritation, lacrimation, burning, and dryness, than people living near their workplace [10]. NO and NO_2_, the primary products of diesel oil consumption by trucks and large vehicles, can travel long distances. A study indicated that higher nitric oxide (NO) and NO_2_ concentrations were related to more severe conjunctivitis in people in Paris [24]. Another study from Taiwan reported that visiting an ophthalmologic outpatient clinic was associated with an increased chance of visiting an ophthalmology clinic for nonspecific conjunctivitis due to increased exposure to PM_10_ and PM_2.5_, NO_2_, SO_2_, and O_3_ [25]. Conjunctival disease caused by air pollution can manifest as subclinical ocular surface changes [55] that frequently cause major discomfort, such as burning and grittiness, and require a clinical visit. Moreover, persistent exposure to air pollution can result in cellular transformation, including goblet-cell hyperplasia in the human conjunctival epithelium [56]. Discomfort due to eye disorders can disrupt people’s daily work efficiency and reduce road traffic safety.

In addition, air pollution may exacerbate dry eye disease [57]. Dysfunction of the tear film is considered to be caused by two interrelated mechanisms: hyperosmolarity and tear film instability [58]. Tear hyperosmolarity may lead to changes on the ocular surface by inducing a series of inflammatory events in the ocular epithelium, which induce the expression of inflammatory mediators in the tear film. Consequent damage to the epithelium causes cell death via apoptosis, loss of goblet cells and decreased mucin production, leading to tear film instability. This instability subsequently disrupts the hyperosmolarity of the ocular surface, intensifying a vicious cycle [59]. A study induced dry eye syndrome in mice with PM_2.5_ eyedrops, and apoptosis of corneal superficial and basal epithelial cells was observed [60].

#### 3.1.2. Glaucoma

Among air pollutants, PM_2.5_ is one of the strongest and most consistent predictors of mortality. It is associated with pulmonary and cardiovascular disease and central nervous system conditions such as Alzheimer’s disease, Parkinson’s disease, and stroke [1]. Wang and associates reported that glaucoma was positively related to national levels of PM_2.5_ [18]. PM_2.5_ has been shown to be toxic to intraocular tissues and contribute to the development of ocular hypertension and glaucoma. Mechanistically, PM_2.5_ and PM_10_ induce the production of NO and interleukin 8, causing increased oxidative stress [61]. Furthermore, PM_2.5_ also increased oxidative stress and induced NLRP3 inflammasome-mediated pyroptosis, a form of nonapoptotic cell death in trabecular meshwork cells, in an in vitro study [62]. Another study also reported that PM_2.5_ exposure inhibited the proliferation of and increased apoptosis in neural retina cells, resulting in the abnormal development of the neural retina [63].

According to the results of a UK Biobank study, participants in areas with higher PM_2.5_ concentrations were more likely to report a diagnosis of glaucoma and to have a thinner macular ganglion cell–inner plexiform layer (GCIPL), as measured by spectral-domain optical coherence tomography (SD-OCT), than their counterparts [19]. There was no association between intraocular pressure (IOP) and PM_2.5_ exposure_,_ suggesting that the relationship may occur through a pressure-independent mechanism, possibly neurotoxic and/or vascular effects [19]. Sun and associates designed a nested case–control study to investigate whether exposure to PM_2.5_ was related to the diagnosis of primary open-angle glaucoma (POAG) in Taiwanese adults [28]. They found that increased exposure to PM_2.5_ was associated with the incidence of POAG. In a retrospective cohort study, Min and colleagues evaluated whether exposure to PM_10_ was related to the occurrence of childhood glaucoma [30]. Their results demonstrated that short-term and long-term exposure to PM_10_ increased the probability of developing childhood glaucoma. This finding implies that PM_10_ exposure may be a risk factor for childhood glaucoma.

#### 3.1.3. Retinopathy and Maculopathy

Air pollution can induce oxidative stress, activate inflammatory pathways, and increase coagulation [64,65,66]. The retina is susceptible to oxidative stress due to its high consumption of oxygen and high proportion of polyunsaturated fatty acids and exposure to visible light [67]. In addition, oxidative damage increases with age, leading to retinal dysfunction and cell loss. A study reported that epithelial–mesenchymal transition (EMT) and activation downstream of cellular reactive oxygen species (ROS) may be responsible for PM_2.5_-induced dysfunction in retinal cells [68]. As a result, the aging retina is potentially particularly susceptible to damage caused by air pollution. There are studies describing myopic macular degeneration, age-related macular degeneration, and diabetic retinopathy in association with air pollution [26,27,29,69]. In one study, exposure to PM_2.5_ and NOx was reported to increase ocular surface inflammation and retinal inflammation, increasing the risk of developing myopic macular diseases [26]. A UK Biobank study including more than 50,000 people demonstrated that exposure to higher PM_2.5_ and PM_10_ concentrations and more PM_2.5_ absorbance were associated with inner and outer retinal layer thinning [29]. Another 10-year cohort study analyzing links between the national health database and the air quality database showed that chronic exposure to a higher concentration of ambient NO_2_ or CO significantly increased the risk of AMD [21]. One study from Taiwan demonstrated a positive association between diabetic retinopathy and PM ≤ 2.5 and 2.5 to 10 μm in diameter, with odds ratios of 1.29 (1.11–1.50) and 1.37 (1.17–1.61), respectively [69]. In a national cross-sectional study in rural China, Shan et al. enrolled 3111 diabetic patients, 329 of whom had diabetic retinopathy [27]. Their results showed that increased exposure to a high concentration of PM_2.5_ was related to an increased risk of diabetic retinopathy (DR) among diabetic patients in rural China. They postulated that PM could raise glucose levels and induce oxidative stress, inflammation, specific cytokine activity, and endothelial dysfunction, contributing to diabetic retinopathy.

### 3.2. Indoor Air Pollution

Indoor air pollution is associated with indoor tobacco smoking, dissipation of compounds used in building materials and decorations in buildings, cooking with oil and high heat, burning coal or biomass for cooking or heating, using pesticides, etc. [70,71]. Moreover, building products and materials, cleaning products, and consumer products emit many chemically nonreactive volatile organic compounds (VOCs) and biologically reactive compounds, such as formaldehyde (FA) and acrolein.

Several VOCs, especially aldehydes, have low odor thresholds [72]. This influences the perceived indoor air quality and possibly overall sensory symptoms [73]. Therefore, personality factors, such as expectations about odor, anxiety level, or attitude toward health risks, may affect complaints of symptoms [74].

Exposure to high concentrations of indoor air pollutants, for example, concentrations of carbon monoxide at 60 × 10^3^ µg/m^3^ for 30 min or 100 × 10^3^ µg/m^3^ for 15 min, could lead to health effects [75]. Examples of acute effects are exacerbation of allergic symptoms, such as conjunctivitis, rhinitis, atopic dermatitis, and intoxication or death due to short-term exposure to very high concentrations of carbon monoxide [76]. Examples of chronic health effects include cancer and noncancer effects related to VOCs [77], respiratory diseases associated with secondhand tobacco smoke (e.g., chronic obstructive pulmonary disease (COPD)) [78], elevated susceptibility to respiratory infections, and cardiovascular disease [38]. Some pollutants, including tobacco smoke and other combustion products, may exacerbate asthma symptoms [79], whereas FA and other VOCs have been associated with sick building syndrome (SBS) [80].

These indoor air pollutants are reported to be harmful to the human eye, as discussed below.

#### 3.2.1. Ocular Surface Disease

Tobacco smoking affects the ocular surface, resulting in symptoms such as itchiness, redness, and irritation of the eyes. The changes on the ocular surface include alteration of the lipid layer of the tear film, reduced tear secretion, and decreased corneal and conjunctival sensitivity and can cause disorders such as atopic kerato-conjunctivitis and allergic conjunctivitis. Tanisha et al. reported that aldehydes and free radicals released from electronic cigarettes may disturb the stability of the tear film, and vape flavoring may damage the lipid layer through peroxidation. Furthermore, nicotine and acrolein in cigarette vapors cause an inflammatory response in corneal epithelial cells [22,23].

Indoor smoking can cause an increased level of PM_2.5_ that is 10 times higher than that in nonsmoking homes. Long-term exposure to fine PM induced oxidative stress in human corneal epithelial-transformed (HCE) cells and altered the cytokine content of tears; moreover, inflammation of the ocular surface and dry eye syndrome subsequently developed in a mouse model [43]. According to a questionnaire study, 82% of participants with household indoor tobacco smoking exposure reported eye irritation [81]. In addition, a large cross-sectional study including over 14,500 adolescents in France showed that environmental tobacco smoke exposure increased the risk of rhinoconjunctivitis by 20% [45].

Indoor smoke can be produced by other sources, such as cookstoves. For example, a study in Guatemala showed that more than 60% of women who used cookstoves for cooking reported that their eyes were always irritated. However, when these participants were divided into those with exposure to miniature chimney stoves and those with exposure to open stoves, the chimney stove group had less eye irritation than the open stove group [82].

Many in vivo and in vitro studies have focused on outdoor air pollution-induced eye disease; however, other studies have revealed correlations between eye diseases and indoor air pollutants. For example, Vitoux’s in vitro study investigated the cytotoxic and inflammatory responses of the conjunctival cell line WKD exposed to combinations of environmental pollutants, such as air–liquid interface conditions combining low humidity, airflow, and formaldehyde gas, to mimic the inflammatory responses observed in dry eye patients [35]. Furthermore, the in vivo study by Suneel et al. investigated acrolein toxicity in rabbit eyes [31]. The results of Suneel showed that topical or vapor application of acrolein severely injured rabbit eyes and led to a series of ocular pathologies, such as swelling of the eye, ocular surface inflammation, abnormalities, irregular collagen accumulation, and corneal opacity. Additionally, Li et al.’s study evaluated long-term cigarette smoke exposure using both in vivo mice and the in vitro conjunctival cell line HCEC, and their results showed that cigarette smoke stimulates ocular surface changes with dry eye, which may be correlated with inflammation and activation of the NF-κB pathway [44].

#### 3.2.2. Glaucoma

Cigarette smoking is associated with many chronic disorders that have been considered serious global public health problems. However, investigations on the correlation between smoking and ocular disorders are scarce. Mechanistically, cigarette smoke extract (CSE) caused injury in primary rat retinal ganglion cells (RGCs) via apoptosis and autophagy by upregulating the mRNA levels of proapoptotic Bad and Bax and the protein level of the autophagy marker LC3B II [33]. This mechanism contributes to the development and progression of glaucoma [34].

Blue Mountains Eye Study data implied a moderate positive association between smoking and elevated IOP (a significant risk factor for glaucoma) [83]. A systematic review to evaluate the association between cigarette smoking and POAG included 17 papers in the final analysis. Their results showed that the link between current smoking and POAG was stronger than that between past smoking and POAG, and recent studies have implied that heavy smoking may elevate the risk of POAG [84]. In a prospective and dynamic cohort study, Pérez-de-Arcelus et al. enrolled 16,797 participants without glaucoma and followed them for a median of 8.5 years. In the 8.5-year follow-up period, 184 new glaucoma cases were diagnosed. Current smoking was associated with a higher glaucoma incidence than never smoking [85].

#### 3.2.3. Cataract

Cataracts have long been linked to cigarette smoking [86]. The mechanism may be direct or indirect effects of inhaled toxic substances on lens tissues. A study enrolled 3924 subjects in rural southern India to investigate the impact of tobacco use on cataract formation; the results demonstrated that cataract formation was significantly associated with tobacco use [20]. Although the exact compounds in cigarettes responsible for lens toxicity are unknown, one compound, naphthalene, is known to be cataractogenic and is used to induce cataracts in rat models [36,37]. Naphthalene, along with another metal toxin, Pb, is also found in biomass fuel (BMF) smoke. The production of smoke during the consumption of this fuel can cause cataract formation [87]. A systematic review of the impact of tobacco smoking on the pathogenesis of many disorders of the anterior segment of the eye in adults and children reported that smoking was a strong risk factor for age-related nuclear cataracts [88].

One-third of the world’s population burns organic material, including wood, feces, or charcoal (BMF), for cooking, heating, and lighting. This form of energy usage is related to high levels of indoor air pollution and an increase in the incidence of respiratory diseases, cardiovascular disorders, and cataracts [89]. Epidemiological research from India and Nepal has shown that indoor cooking using BMF is related to cataracts and blindness [90,91]. Smoke causes oxidative stress and consumes plasma ascorbate, carotenoids, and glutathione, which offer antioxidant protection against cataract formation.

#### 3.2.4. Uveitis

As uveitis develops due to immune dysregulation, data on the association between smoking and uveitis are rare. An epidemiological study in the American adult population using data from the National Health and Nutrition Examination Survey (NHANES) for 2009 and 2010 demonstrated that smoking was positively associated with uveitis [92]. The Pacific Ocular Inflammation Study implied that cigarette smoking was significantly associated with new-onset uveitis [93]. There is a stronger association between smoking and noninfectious uveitis. In a retrospective case–control study, Lin and associates reported that smoking was related to both infectious and noninfectious uveitis [94]. An observational cross-sectional study enrolled 350 patients with noninfectious uveitis. Roesel and colleagues found that smoking had a positive association with uveitis activity, resulting in an increased dose of steroid eye drops and increased occurrence of cataract and macular edema [95].

Chronic exposure to ROS in cigarette smoke upregulates the expression of TLR4 by human macrophages, promoting NF-κB activation and the production of interleukin (IL)-8 [96,97]. Nicotine plays a similar role in neutrophils by generating peroxynitrite, a nitrate isomer that binds acetylcholine receptors to promote NF-κB-mediated cytokine IL-8 transcription [39,97]. Increased concentrations of IL-8, as found in the aqueous humor (AqH) in uveitis, act together with IL-6 and TNF-α to facilitate the migration and activation of macrophages that assault the uvea [40,41,42].

Several carcinogenic compounds cause Th17 cell expansion by binding aryl hydrocarbon receptors on memory T cells [98,99]. The resultant increase in the Th17 population results in the elevated secretion of IL-17 and IL-22, which conversely facilitate the migration and extravasation of leukocytes into various tissues [100]. Updated data have thus suggested that Th17 cells play a role in the pathological mechanism of not only uveitis but also multiple sclerosis, rheumatoid arthritis, and psoriasis [100,101,102,103,104,105]. This shared pathogenesis perhaps illustrates why smoking is related to several and often concomitantly occurring autoimmune disorders.

#### 3.2.5. Retinal and Macular Diseases

Previous studies have shown that cigarette smoking is related to an increased risk of AMD [106]. Regarding environmental cigarette smoke exposure, a case–control study showed increased risks of neovascular and atrophic AMD [107]. However, in the Blue Mountains Eye Study, Smith and associates found that passive smoking does not significantly increase the risk for late AMD [108]. Cigarette vapor was reported to accelerate the progression of inflammation and angiogenesis in the retina of mice, which were possibly associated with the onset of wet AMD [109]. In addition, nicotine inhaled in passive smoking increased the VEGF-to-PEDF ratio in RPE cells in an in vitro study. This alteration in the ratio may play a key role in the progression to wet AMD in passive smokers [110].

A systematic review of the impact of direct tobacco smoking on the pathogenesis of many disorders of the posterior segment of the eye in adults and children revealed that tobacco smoking had a positive association with AMD, polypoidal choroidal vasculopathy, and inflamed cystoid macular edema in adults. Tobacco smoking decreases retinal and choroidal thickness. In addition, maternal smoking is a significant risk factor for stage 3 and 4 retinopathy of prematurity and a thinner retinal nerve fiber layer in children [111]. Govindaraju et al. reported cigarette smoke-induced proteostasis and autophagy impairment, which may be associated with AMD pathogenesis, in retinal pigmental ARPE-19 cells [32].

Household fuel consumption is related to an increased indoor concentration of fine particles. Exposure to fine particles associated with solid fuel use causes systemic inflammatory responses and cytokine production. To our knowledge, AMD is due to genetic polymorphisms, and innate immune reactions and inflammation are recognized as etiologies. Although there is a lack of evidence, chronic inflammation induced by indoor air pollution is a possible cause of AMD [70].

## 4. Conclusions

Outdoor and indoor air pollution is derived from different sources and can cause different eye diseases. Ocular surface irrigation, conjunctivitis and dry eye disease are the most direct results of air pollution. However, chronic inflammation, oxidative stress, and toxicity resulting from air pollution can further cause cataracts, glaucoma, uveitis, retinal layer thinning, macular degeneration, and diabetic retinopathy. Further research on the effects of air pollution on retinal ganglion cells and the chorioretinal vasculature may help identify the underlying pathological mechanisms. In addition, further research on the association between air pollutants and ophthalmological disorders is needed to improve the understanding of exposure patterns and ocular effects. Such studies will help determine the long-term impacts of air pollutants on the eye, which are currently unknown.

## Figures and Tables

**Table 1:** Eye-damaging air pollutants and their relevant studies on eye diseases.

Pollutant(s)	Ocular Disorder(s)	Study Model	Reference
Outdoor pollutant(s)			
CO	AMD	Population-based study	[21]
NO	Conjunctivitis	Data collection	[22,23]
NO_2_	AMD	Population-based study	[21]
	Conjunctivitis	Data collection	[24]
	Nonspecific conjunctivitis		[25]
NO_x_	Myopia	Data collection	[26]
		In vivo/hamster model	
O_3_	Nonspecific conjunctivitis	Data collection	[25]
PM_2.5_	DR	National cross-sectional study	[27]
	Myopia	Data collection	[26]
		In vivo/hamster model	
	Nonspecific conjunctivitis	Data collection	[25]
	POAG	Nested case–control study	[28]
	Retinal layer thinning	Community-based cohort study	[29]
PM_10_	Childhood glaucoma	Retrospective cohort study	[30]
	Nonspecific conjunctivitis	Data collection	[25]
	Retinal layer thinning	Community-based cohort study	[29]
SO_2_	Nonspecific conjunctivitis	Data collection	[25]
Indoor pollutant(s)			
Acrolein	OSD	In vivo/rabbit model	[31]
Aldehydes	Tear film instability and lipid layer peroxidation	Tears from subjects	[22,23]
CSE	AMD	In vitro/ARPE-19 cells	[32]
	Glaucoma	In vitro/primary rat RGCs	[33,34]
Formaldehyde	OSD	In vitro/WKD cells	[35]
Naphthalene	Cataracts	In vivo/rat model	[36,37]
Nicotine	OSD	In vitro/corneal epithelial cells	[38]
	Uveitis	Subjects’ and patients’ AqH	[39,40,41,42]
PM_2.5_	Dry eye syndrome	In vitro/HCE cells	[43]
		In vivo/mouse model	
Tabaco smoking	OSD	In vitro/HCE cells	[44]
		In vivo/mouse model	
	Rhino-conjunctivitis	Cross-sectional study	[45]

**Table 2:** WHO 2021 air pollution guidelines in comparison with guidelines in 2005.

Pollutant(s)	Averaging Time	WHO 2021 Air Quality Guideline	WHO 2005 Air Quality Guideline	Change
PM_2.5_ (μg/m^3^)	Annual	5	10	−50%
	24-h	15	25	−40%
PM_10_ (μg/m^3^)	Annual	15	20	−25%
	24-h	45	50	−10%
O_3_ (μg/m^3^)	Peak season	60	N/A	Newly introduced
	8-h	100	100	Unchanged
NO_2_ (μg/m^3^)	Annual	10	40	−75%
	24-h	25	N/A	Newly introduced
	1-h	200	200	Unchanged
SO_2_ (μg/m^3^)	24-h	40	20	+100%
	10-min	500	500	Unchanged
CO (mg/m^3^)	24-h	4	N/A	Newly introduced
	8-h	10	N/A	Newly introduced
	1-h	35	N/A	Newly introduced
	15-min	100	N/A	Newly introduced

## Data Availability

Not applicable.

## References

[1] Cohen A.J., Brauer M., Burnett R., Anderson H.R., Frostad J., Estep K., Balakrishnan K., Brunekreef B., Dandona L., Dandona R. (2017). Estimates and 25-year trends of the global burden of disease attributable to ambient air pollution: An analysis of data from the Global Burden of Diseases Study 2015. Lancet.

[2] Di Q., Wang Y., Zanobetti A., Wang Y., Koutrakis P., Choirat C., Dominici F., Schwartz J.D. (2017). Air Pollution and Mortality in the Medicare Population. N. Engl. J. Med..

[3] Abellán R., Mansego M.L., Martínez-Hervás S., Martín-Escudero J.C., Carmena R., Real J.T., Redon J., Castrodeza-Sanz J.J., Chaves F.J. (2010). Association of selected ABC gene family single nucleotide polymorphisms with postprandial lipoproteins: Results from the population-based Hortega study. Atherosclerosis.

[4] Kemp A.C., Horton B.P., Donnelly J.P., Mann M.E., Vermeer M., Rahmstorf S. (2011). Climate related sea-level variations over the past two millennia. Proc. Natl. Acad. Sci. USA.

[5] Swenberg J.A., Moeller B.C., Lu K., Rager J.E., Fry R.C., Starr T.B. (2013). Formaldehyde carcinogenicity research: 30 years and counting for mode of action, epidemiology, and cancer risk assessment. Toxicol. Pathol..

[6] Zhang J., Smith K.R. (2003). Indoor air pollution: A global health concern. Br. Med. Bull..

[7] Eftim S.E., Samet J.M., Janes H., McDermott A., Dominici F. (2008). Fine particulate matter and mortality: A comparison of the six cities and American Cancer Society cohorts with a medicare cohort. Epidemiology.

[8] Pope C.A. (2004). Air pollution and health-good news and bad. N. Engl. J. Med..

[9] Pope C.A., Dockery D.W. (2006). Health effects of fine particulate air pollution: Lines that connect. J. Air. Waste Manag. Assoc..

[10] Saxena R., Srivastava S., Trivedi D., Anand E., Joshi S., Gupta S.K. (2003). Impact of environmental pollution on the eye. Acta Ophthalmol. Scand..

[11] Tuominen I.S., Konttinen Y.T., Vesaluoma M.H., Moilanen J.A., Helintö M., Tervo T.M. (2003). Corneal innervation and morphology in primary Sjögren’s syndrome. Invest. Ophthalmol. Vis. Sci..

[12] Al-Aqaba M.A., Dhillon V.K., Mohammed I., Said D.G., Dua H.S. (2019). Corneal nerves in health and disease. Prog. Retin. Eye Res..

[13] Koh S., Tung C.I., Inoue Y., Jhanji V. (2018). Effects of tear film dynamics on quality of vision. Br. J. Ophthalmol..

[14] Schwela D. (2000). Air pollution and health in urban areas. Rev. Environ. Health.

[15] Versura P., Profazio V., Cellini M., Torreggiani A., Caramazza R. (1999). Eye discomfort and air pollution. Ophthalmologica.

[16] Mimura T., Ichinose T., Yamagami S., Fujishima H., Kamei Y., Goto M., Takada S., Matsubara M. (2014). Airborne particulate matter (PM2.5) and the prevalence of allergic conjunctivitis in Japan. Sci. Total Environ..

[17] Hong J., Zhong T., Li H., Xu J., Ye X., Mu Z., Lu Y., Mashaghi A., Zhou Y., Tan M. (2016). Ambient air pollution, weather changes, and outpatient visits for allergic conjunctivitis: A retrospective registry study. Sci. Rep..

[18] Wang W., He M., Li Z., Huang W. (2019). Epidemiological variations and trends in health burden of glaucoma worldwide. Acta Ophthalmol..

[19] Chua S.Y.L., Khawaja A.P., Morgan J., Strouthidis N., Reisman C., Dick A.D., Khaw P.T., Patel P.J., Foster P.J. (2019). The Relationship Between Ambient Atmospheric Fine Particulate Matter (PM2.5) and Glaucoma in a Large Community Cohort. Invest Ophthalmol. Vis. Sci..

[20] Raju P., George R., Ve Ramesh S., Arvind H., Baskaran M., Vijaya L. (2006). Influence of tobacco use on cataract development. Br. J. Ophthalmol..

[21] Chang K.-H., Hsu P.-Y., Lin C.-J., Lin C.-L., Juo S.-H.H., Liang C.-L. (2019). Traffic-related air pollutants increase the risk for age-related macular degeneration. J. Investig. Med..

[22] Jetten N., Verbruggen S., Gijbels M.J., Post M.J., De Winther M.P., Donners M.M. (2014). Anti-inflammatory M2, but not pro-inflammatory M1 macrophages promote angiogenesis in vivo. Angiogenesis.

[23] Ambient (Outdoor) Air Quality and Health. https://www.who.int/mediacentre/factsheets/fs313/en/.

[24] Aitio A., Axelson O., Blair A., Bond J.A., Bucher J.R., Caldwell J., Coggon D., Demers P.A., Dragani T.A., Elcombe C.R. (2016). Outdoor Air Pollution. IARC Monogr. Eval. Carcinog. Risks Hum..

[25] Huff R.D., Carlsten C., Hirota J.A. (2019). An update on immunologic mechanisms in the respiratory mucosa in response to air pollutants. J. Allergy Clin. Immunol..

[26] Kappos A.D., Bruckmann P., Eikmann T., Englert N., Heinrich U., Höppe P., Koch E., Krause G.H., Kreyling W.G., Rauchfuss K. (2004). Health effects of particles in ambient air. Int. J. Hyg. Environ. Health.

[27] Holguin F. (2008). Traffic, outdoor air pollution, and asthma. Immunol. Allergy Clin. N. Am..

[28] Hetland R.B., Cassee F.R., Låg M., Refsnes M., Dybing E., Schwarze P.E. (2005). Cytokine release from alveolar macrophages exposed to ambient particulate matter: Heterogeneity in relation to size, city and season. Part. Fibre Toxicol..

[29] McCreanor J., Cullinan P., Nieuwenhuijsen M.J., Stewart-Evans J., Malliarou E., Jarup L., Harrington R., Svartengren M., Han I.K., Ohman-Strickland P. (2007). Respiratory effects of exposure to diesel traffic in persons with asthma. N. Engl. J. Med..

[30] Vohra K., Vodonos A., Schwartz J., Marais E.A., Sulprizio M.P., Mickley L.J. (2021). Global mortality from outdoor fine particle pollution generated by fossil fuel combustion: Results from GEOS-Chem. Environ. Res..

[31] Bourcier T., Viboud C., Cohen J.C., Thomas F., Bury T., Cadiot L., Mestre O., Flahault A., Borderie V., Laroche L. (2003). Effects of air pollution and climatic conditions on the frequency of ophthalmological emergency examinations. Br. J. Ophthalmol..

[32] Chang C.J., Yang H.H., Chang C.A., Tsai H.Y. (2012). Relationship between air pollution and outpatient visits for nonspecific conjunctivitis. Invest. Ophthalmol. Vis. Sci..

[33] Torricelli A., Novaes P., Matsuda M., Alves M., Monteiro M. (2011). Ocular surface adverse effects of ambient levels of air pollution. Arq. Bras. De Oftalmol..

[34] Novaes P., do Nascimento Saldiva P.H., Kara-José N., Macchione M., Matsuda M., Racca L., Berra A. (2007). Ambient levels of air pollution induce goblet-cell hyperplasia in human conjunctival epithelium. Environ. Health Perspect..

[35] Zhong J.-Y., Lee Y.-C., Hsieh C.-J., Tseng C.-C., Yiin L.-M. (2018). Association between Dry Eye Disease, Air Pollution and Weather Changes in Taiwan. Int. J. Environ. Res. Public Health.

[36] Liu H., Begley C., Chen M., Bradley A., Bonanno J., McNamara N.A., Nelson J.D., Simpson T. (2009). A link between tear instability and hyperosmolarity in dry eye. Invest. Ophthalmol. Vis. Sci..

[37] Bron A.J., Abelson M.B., Ousler G., Pearce E., Tomlinson A., Yokoi A., Smith J.A., Begley C., Caffery B., Nichols K. (2007). Methodologies to diagnose and monitor dry eye disease: Report of the Diagnostic Methodology Subcommittee of the International Dry Eye WorkShop (2007). Ocul. Surf..

[38] Tan G., Li J., Yang Q., Wu A., Qu D.Y., Wang Y., Ye L., Bao J., Shao Y. (2018). Air pollutant particulate matter 2.5 induces dry eye syndrome in mice. Sci. Rep..

[39] Yoon S., Han S., Jeon K.J., Kwon S. (2018). Effects of collected road dusts on cell viability, inflammatory response, and oxidative stress in cultured human corneal epithelial cells. Toxicol. Lett..

[40] Li L., Xing C., Zhou J., Niu L., Luo B., Song M., Niu J., Ruan Y., Sun X., Lei Y. (2021). Airborne particulate matter (PM_2.5_) triggers ocular hypertension and glaucoma through pyroptosis. Part. Fibre Toxicol..

[41] Zeng Y., Li M., Zou T., Chen X., Li Q., Li Y., Ge L., Chen S., Xu H. (2021). The Impact of Particulate Matter (PM_2.5_) on Human Retinal Development in hESC-Derived Retinal Organoids. Front. Cell Dev. Biol..

[42] Sun H.Y., Luo C.W., Chiang Y.W., Yeh K.L., Li Y.C., Ho Y.C., Lee S.S., Chen W.Y., Chen C.J., Kuan Y.H. (2021). Association Between PM_2.5_ Exposure Level and Primary Open-Angle Glaucoma in Taiwanese Adults: A Nested Case-Control Study. Int. J. Environ. Res. Public Health.

[43] Min K.B., Min J.Y. (2020). Association of Ambient Particulate Matter Exposure with the Incidence of Glaucoma in Childhood. Am. J. Ophthalmol..

[44] Lodovici M., Bigagli E. (2011). Oxidative stress and air pollution exposure. J. Toxicol..

[45] Baccarelli A., Zanobetti A., Martinelli I., Grillo P., Hou L., Giacomini S., Bonzini M., Lanzani G., Mannucci P.M., Bertazzi P.A. (2007). Effects of exposure to air pollution on blood coagulation. J. Thromb. Haemost..

[46] Brook R.D., Rajagopalan S., Pope C.A., Brook J.R., Bhatnagar A., Diez-Roux A.V., Holguin F., Hong Y., Luepker R.V., Mittleman M.A. (2010). Particulate matter air pollution and cardiovascular disease: An update to the scientific statement from the American Heart Association. Circulation.

[47] Jarrett S.G., Boulton M.E. (2012). Consequences of oxidative stress in age-related macular degeneration. Mol. Asp. Med..

[48] Lee H., Hwang-Bo H., Ji S.Y., Kim M.Y., Kim S.Y., Park C., Hong S.H., Kim G.Y., Song K.S., Hyun J.W. (2020). Diesel particulate matter2.5 promotes epithelial-mesenchymal transition of human retinal pigment epithelial cells via generation of reactive oxygen species. Environ. Pollut..

[49] Wei C.C., Lin H.J., Lim Y.P., Chen C.S., Chang C.Y., Lin C.J., Chen J.J., Tien P.T., Lin C.L., Wan L. (2019). PM_2.5_ and NOx exposure promote myopia: Clinical evidence and experimental proof. Environ. Pollut..

[50] Chua S.Y.L., Khawaja A.P., Dick A.D., Morgan J., Dhillon B., Lotery A.J., Strouthidis N.G., Reisman C., Peto T., Khaw P.T. (2020). Ambient Air Pollution Associations with Retinal Morphology in the UK Biobank. Investig. Ophthalmol. Vis. Sci..

[51] Pan S.C., Huang C.C., Chin W.S., Chen B.Y., Chan C.C., Guo Y.L. (2020). Association between air pollution exposure and diabetic retinopathy among diabetics. Environ. Res..

[52] Shan A., Chen X., Yang X., Yao B., Liang F., Yang Z., Liu F., Chen S., Yan X., Huang J. (2021). Association between long-term exposure to fine particulate matter and diabetic retinopathy among diabetic patients: A national cross-sectional study in China. Environ. Int..

[53] West S.K., Bates M.N., Lee J.S., Schaumberg D.A., Lee D.J., Adair-Rohani H., Chen D.F., Araj H. (2013). Is household air pollution a risk factor for eye disease?. Int. J. Environ. Res. Public Health.

[54] Kabir E., Kim K.H. (2011). An investigation on hazardous and odorous pollutant emission during cooking activities. J. Hazard. Mater..

[55] Wolkoff P. (2017). External eye symptoms in indoor environments. Indoor Air.

[56] Wolkoff P. (2013). Indoor air pollutants in office environments: Assessment of comfort, health, and performance. Int. J. Hyg. Environ. Health.

[57] Jaén C., Dalton P. (2014). Asthma and odors: The role of risk perception in asthma exacerbation. J. Psychosom. Res..

[58] Luengas A., Barona A., Hort C., Gallastegui G., Platel V., Elias A. (2015). A review of indoor air treatment technologies. Rev. Environ. Sci. Bio/Technol..

[59] de Juniac A., Kreis I., Ibison J., Murray V. (2012). Epidemiology of unintentional carbon monoxide fatalities in the UK. Int. J. Environ. Health Res..

[60] Sarigiannis D.A., Karakitsios S.P., Gotti A., Liakos I.L., Katsoyiannis A. (2011). Exposure to major volatile organic compounds and carbonyls in European indoor environments and associated health risk. Environ. Int..

[61] Jordan R.E., Cheng K.K., Miller M.R., Adab P. (2011). Passive smoking and chronic obstructive pulmonary disease: Cross-sectional analysis of data from the Health Survey for England. BMJ Open.

[62] Blanc P.D., Eisner M.D., Katz P.P., Yen I.H., Archea C., Earnest G., Janson S., Masharani U.B., Quinlan P.J., Hammond S.K. (2005). Impact of the home indoor environment on adult asthma and rhinitis. J. Occup. Environ. Med..

[63] Rushton L. (2004). Health impact of environmental tobacco smoke in the home. Rev. Environ. Health.

[64] Gawande S., Tiwari R.R., Narayanan P., Bhadri A. (2020). Indoor Air Quality and Sick Building Syndrome: Are Green Buildings Better than Conventional Buildings?. Indian J. Occup. Environ. Med..

[65] Martheswaran T., Shmunes M.H., Ronquillo Y.C., Moshirfar M. (2021). The impact of vaping on ocular health: A literature review. Int. Ophthalmol..

[66] Choi W., Lian C., Ying L., Kim G.E., You I.C., Park S.H., Yoon K.C. (2016). Expression of Lipid Peroxidation Markers in the Tear Film and Ocular Surface of Patients with Non-Sjogren Syndrome: Potential Biomarkers for Dry Eye Disease. Curr. Eye Res..

[67] Yang Q., Li K., Li D., Zhang Y., Liu X., Wu K. (2019). Effects of fine particulate matter on the ocular surface: An in vitro and in vivo study. Biomed. Pharmacother..

[68] Bascom R., Kulle T., Kagey-Sobotka A., Proud D. (1991). Upper respiratory tract environmental tobacco smoke sensitivity. Am. Rev. Respir. Dis..

[69] Annesi-Maesano I., Oryszczyn M.P., Raherison C., Kopferschmitt C., Pauli G., Taytard A., Tunon de Lara M., Vervloet D., Charpin D. (2004). Increased prevalence of asthma and allied diseases among active adolescent tobacco smokers after controlling for passive smoking exposure. A cause for concern?. Clin. Exp. Allergy.

[70] Díaz E., Smith-Sivertsen T., Pope D., Lie R.T., Díaz A., McCracken J., Arana B., Smith K.R., Bruce N. (2007). Eye discomfort, headache and back pain among Mayan Guatemalan women taking part in a randomised stove intervention trial. J. Epidemiol. Community Health.

[71] Vitoux M.A., Kessal K., Baudouin C., Laprévote O., Melik Parsadaniantz S., Achard S., Brignole-Baudouin F. (2018). Formaldehyde Gas Exposure Increases Inflammation in an In Vitro Model of Dry Eye. Toxicol. Sci..

[72] Gupta S., Fink M.K., Martin L.M., Sinha P.R., Rodier J.T., Sinha N.R., Hesemann N.P., Chaurasia S.S., Mohan R.R. (2020). A rabbit model for evaluating ocular damage from acrolein toxicity in vivo. Ann. N. Y. Acad. Sci..

[73] Li J., Zhang G., Nian S., Lv Y., Shao Y., Qiao N., Liang R., Huang L., Luo A. (2020). Dry eye induced by exposure to cigarette smoke pollution: An in vivo and in vitro study. Free. Radic. Biol. Med..

[74] Lee K., Hong S., Seong G.J., Kim C.Y. (2016). Cigarette Smoke Extract Causes Injury in Primary Retinal Ganglion Cells via Apoptosis and Autophagy. Curr. Eye Res..

[75] Smith C.A., Vianna J.R., Chauhan B.C. (2017). Assessing retinal ganglion cell damage. Eye.

[76] Lee A.J., Rochtchina E., Wang J.J., Healey P.R., Mitchell P. (2003). Does smoking affect intraocular pressure? Findings from the Blue Mountains Eye Study. J. Glaucoma.

[77] Jain V., Jain M., Abdull M.M., Bastawrous A. (2017). The association between cigarette smoking and primary open-angle glaucoma: A systematic review. Int. Ophthalmol..

[78] Pérez-de-Arcelus M., Toledo E., Martínez-González M., Martín-Calvo N., Fernández-Montero A., Moreno-Montañés J. (2017). Smoking and incidence of glaucoma: The SUN Cohort. Medicine.

[79] (2004). Reports of the Surgeon General. The Health Consequences of Smoking: A Report of the Surgeon General.

[80] Nagata M., Kojima M., Sasaki K. (1999). Effect of vitamin E eye drops on naphthalene-induced cataract in rats. J. Ocul. Pharmacol. Ther..

[81] Xu G.T., Zigler J.S., Lou M.F. (1992). The possible mechanism of naphthalene cataract in rat and its prevention by an aldose reductase inhibitor (ALO1576). Exp. Eye Res..

[82] Schaumberg D.A., Mendes F., Balaram M., Dana M.R., Sparrow D., Hu H. (2004). Accumulated lead exposure and risk of age-related cataract in men. JAMA.

[83] Nita M., Grzybowski A. (2017). Smoking and Eye Pathologies. A Systemic Review. Part I. Anterior Eye Segment Pathologies. Curr. Pharm. Des..

[84] Fullerton D.G., Bruce N., Gordon S.B. (2008). Indoor air pollution from biomass fuel smoke is a major health concern in the developing world. Trans. R. Soc. Trop. Med. Hyg..

[85] Saha A., Kulkarni P.K., Shah A., Patel M., Saiyed H.N. (2005). Ocular morbidity and fuel use: An experience from India. Occup. Environ. Med..

[86] Pokhrel A.K., Smith K.R., Khalakdina A., Deuja A., Bates M.N. (2005). Case-control study of indoor cooking smoke exposure and cataract in Nepal and India. Int. J. Epidemiol..

[87] González M.M., Solano M.M., Porco T.C., Oldenburg C.E., Acharya N.R., Lin S.C., Chan M.F. (2018). Epidemiology of uveitis in a US population-based study. J. Ophthalmic Inflamm. Infect..

[88] Yuen B.G., Tham V.M., Browne E.N., Weinrib R., Borkar D.S., Parker J.V., Uchida A., Vinoya A.C., Acharya N.R. (2015). Association between Smoking and Uveitis: Results from the Pacific Ocular Inflammation Study. Ophthalmology.

[89] Lin P., Loh A.R., Margolis T.P., Acharya N.R. (2010). Cigarette smoking as a risk factor for uveitis. Ophthalmology.

[90] Roesel M., Ruttig A., Schumacher C., Heinz C., Heiligenhaus A. (2011). Smoking complicates the course of non-infectious uveitis. Graefe’s Arch. Clin. Exp. Ophthalmol..

[91] Sarir H., Mortaz E., Karimi K., Kraneveld A.D., Rahman I., Caldenhoven E., Nijkamp F.P., Folkerts G. (2009). Cigarette smoke regulates the expression of TLR4 and IL-8 production by human macrophages. J. Inflamm..

[92] Gonçalves R.B., Coletta R.D., Silvério K.G., Benevides L., Casati M.Z., da Silva J.S., Nociti F.H. (2011). Impact of smoking on inflammation: Overview of molecular mechanisms. Inflamm. Res..

[93] Iho S., Tanaka Y., Takauji R., Kobayashi C., Muramatsu I., Iwasaki H., Nakamura K., Sasaki Y., Nakao K., Takahashi T. (2003). Nicotine induces human neutrophils to produce IL-8 through the generation of peroxynitrite and subsequent activation of NF-kappaB. J. Leukoc. Biol..

[94] Curnow S.J., Falciani F., Durrani O.M., Cheung C.M., Ross E.J., Wloka K., Rauz S., Wallace G.R., Salmon M., Murray P.I. (2005). Multiplex bead immunoassay analysis of aqueous humor reveals distinct cytokine profiles in uveitis. Invest. Ophthalmol. Vis. Sci..

[95] Valentincic N.V., de Groot-Mijnes J.D., Kraut A., Korosec P., Hawlina M., Rothova A. (2011). Intraocular and serum cytokine profiles in patients with intermediate uveitis. Mol. Vis..

[96] Khera T.K., Copland D.A., Boldison J., Lait P.J., Szymkowski D.E., Dick A.D., Nicholson L.B. (2012). Tumour necrosis factor-mediated macrophage activation in the target organ is critical for clinical manifestation of uveitis. Clin. Exp. Immunol..

[97] Quintana F.J., Basso A.S., Iglesias A.H., Korn T., Farez M.F., Bettelli E., Caccamo M., Oukka M., Weiner H.L. (2008). Control of T(reg) and T(H)17 cell differentiation by the aryl hydrocarbon receptor. Nature.

[98] Veldhoen M., Hirota K., Westendorf A.M., Buer J., Dumoutier L., Renauld J.C., Stockinger B. (2008). The aryl hydrocarbon receptor links TH17-cell-mediated autoimmunity to environmental toxins. Nature.

[99] Torii K., Saito C., Furuhashi T., Nishioka A., Shintani Y., Kawashima K., Kato H., Morita A. (2011). Tobacco smoke is related to Th17 generation with clinical implications for psoriasis patients. Exp. Dermatol..

[100] Escobar T., Yu C.R., Muljo S.A., Egwuagu C.E. (2013). STAT3 activates miR-155 in Th17 cells and acts in concert to promote experimental autoimmune uveitis. Investig. Ophthalmol. Vis. Sci..

[101] Wang R.X., Yu C.R., Mahdi R.M., Egwuagu C.E. (2012). Novel IL27p28/IL12p40 cytokine suppressed experimental autoimmune uveitis by inhibiting autoreactive Th1/Th17 cells and promoting expansion of regulatory T cells. J. Biol. Chem..

[102] Jadidi-Niaragh F., Mirshafiey A. (2011). Th17 cell, the new player of neuroinflammatory process in multiple sclerosis. Scand. J. Immunol..

[103] van Hamburg J.P., Asmawidjaja P.S., Davelaar N., Mus A.M., Colin E.M., Hazes J.M., Dolhain R.J., Lubberts E. (2011). Th17 cells, but not Th1 cells, from patients with early rheumatoid arthritis are potent inducers of matrix metalloproteinases and proinflammatory cytokines upon synovial fibroblast interaction, including autocrine interleukin-17A production. Arthritis Rheum..

[104] Zhang L., Li J.M., Liu X.G., Ma D.X., Hu N.W., Li Y.G., Li W., Hu Y., Yu S., Qu X. (2011). Elevated Th22 cells correlated with Th17 cells in patients with rheumatoid arthritis. J. Clin. Immunol..

[105] Smith W., Assink J., Klein R., Mitchell P., Klaver C.C., Klein B.E., Hofman A., Jensen S., Wang J.J., de Jong P.T. (2001). Risk factors for age-related macular degeneration: Pooled findings from three continents. Ophthalmology.

[106] Khan J.C., Thurlby D.A., Shahid H., Clayton D.G., Yates J.R., Bradley M., Moore A.T., Bird A.C. (2006). Smoking and age related macular degeneration: The number of pack years of cigarette smoking is a major determinant of risk for both geographic atrophy and choroidal neovascularisation. Br. J. Ophthalmol..

[107] Smith W., Mitchell P., Leeder S.R. (1996). Smoking and age-related maculopathy. The Blue Mountains Eye Study. Arch. Ophthalmol..

[108] Bai Y.C., Wang C.Y., Lin C.L., Lai J.N., Wei J.C. (2021). Association Between Air Pollution and the Risk of Uveitis: A Nationwide, Population-Based Cohort Study. Front. Immunol..

[109] Pons M., Marin-Castano M.E. (2011). Nicotine increases the VEGF/PEDF ratio in retinal pigment epithelium: A possible mechanism for CNV in passive smokers with AMD. Invest. Ophthalmol. Vis. Sci..

[110] Nita M., Grzybowski A. (2017). Smoking and Eye Pathologies. A Systemic Review. Part II. Retina Diseases, Uveitis, Optic Neuropathies, Thyroid-Associated Orbitopathy. Curr. Pharm. Des..

[111] Govindaraju V.K., Bodas M., Vij N. (2017). Cigarette smoke induced autophagy-impairment regulates AMD pathogenesis mechanisms in ARPE-19 cells. PLoS ONE.

